# Hypoxic oligodendrocyte precursor cell-derived VEGFA is associated with blood–brain barrier impairment

**DOI:** 10.1186/s40478-023-01627-5

**Published:** 2023-08-07

**Authors:** Narek Manukjan, Daria Majcher, Peter Leenders, Florian Caiment, Marcel van Herwijnen, Hubert J. Smeets, Ernst Suidgeest, Louise van der Weerd, Tim Vanmierlo, Jacobus F. A. Jansen, Walter H. Backes, Robert J. van Oostenbrugge, Julie Staals, Daniel Fulton, Zubair Ahmed, W. Matthijs Blankesteijn, Sébastien Foulquier

**Affiliations:** 1 Department of Pharmacology and Toxicology, Maastricht University, P.O. Box 616, 6200 MD Maastricht, The Netherlands; 2 CARIM - School for Cardiovascular Diseases, Maastricht University, P.O. Box 616, 6200 MD Maastricht, The Netherlands; 3 Neuroscience and Ophthalmology, Institute of Inflammation and Ageing, University of Birmingham, Edgbaston, Birmingham, B15 2TT UK; 4 Department of Toxicogenomics, GROW–School for Oncology and Developmental Biology, Maastricht University, P.O. Box 616, 6200 MD Maastricht, The Netherlands; 5 MHeNs—School for Mental Health and Neuroscience, Maastricht University, P.O. Box 616, 6200 MD Maastricht, The Netherlands; 6 C.J. Gorter Center for High Field MRI, Department of Radiology, Leiden University Medical Center, P.O. Box 9500, 2300 RA Leiden, the Netherlands; 7 Department of Human Genetics, Leiden University Medical Center, P.O. Box 9500, 2300 RA Leiden, The Netherlands; 8 Department of Neuroscience, Biomedical Research Institute, Hasselt University, 3500 Hasselt, Belgium; 9 Department of Psychiatry and Neuropsychology, European Graduate School of Neuroscience, Maastricht University, P.O. Box 616, 6200 MD Maastricht, The Netherlands; 10 Department of Radiology and Nuclear Medicine, Maastricht University Medical Center+, P.O. Box 5800, 6202 AZ Maastricht, The Netherlands; 11 Department of Neurology, Maastricht University Medical Center+, P.O. Box 5800, 6202 AZ Maastricht, The Netherlands; 12 Centre for Trauma Sciences Research, University of Birmingham, Edgbaston, Birmingham, B15 2TT UK

**Keywords:** Vascular dementia, Glial biology, OPC, Angiogenesis, BBB, cSVD

## Abstract

Cerebral small vessel disease is characterised by decreased cerebral blood flow and blood–brain barrier impairments which play a key role in the development of white matter lesions. We hypothesised that cerebral hypoperfusion causes local hypoxia, affecting oligodendrocyte precursor cell—endothelial cell signalling leading to blood–brain barrier dysfunction as an early mechanism for the development of white matter lesions. Bilateral carotid artery stenosis was used as a mouse model for cerebral hypoperfusion. Pimonidazole, a hypoxic cell marker, was injected prior to humane sacrifice at day 7. Myelin content, vascular density, blood–brain barrier leakages, and hypoxic cell density were quantified. Primary mouse oligodendrocyte precursor cells were exposed to hypoxia and RNA sequencing was performed. *Vegfa* gene expression and protein secretion was examined in an oligodendrocyte precursor cell line exposed to hypoxia. Additionally, human blood plasma VEGFA levels were measured and correlated to blood–brain barrier permeability in normal-appearing white matter and white matter lesions of cerebral small vessel disease patients and controls. Cerebral blood flow was reduced in the stenosis mice, with an increase in hypoxic cell number and blood–brain barrier leakages in the cortical areas but no changes in myelin content or vascular density. *Vegfa* upregulation was identified in hypoxic oligodendrocyte precursor cells, which was mediated via *Hif1α* and *Epas1*. In humans, VEGFA plasma levels were increased in patients versus controls. VEGFA plasma levels were associated with increased blood–brain barrier permeability in normal appearing white matter of patients. Cerebral hypoperfusion mediates hypoxia induced VEGFA expression in oligodendrocyte precursor cells through *Hif1α/Epas1* signalling. VEGFA could in turn increase BBB permeability. In humans, increased VEGFA plasma levels in cerebral small vessel disease patients were associated with increased blood–brain barrier permeability in the normal appearing white matter. Our results support a role of VEGFA expression in cerebral hypoperfusion as seen in cerebral small vessel disease.

**Supplementary Information:**

The online version contains supplementary material available at 10.1186/s40478-023-01627-5.

## Introduction

Cerebral small vessel disease (cSVD) is an umbrella term referring to pathological processes affecting the small arteries, arterioles, venules, and capillaries in the brain, leading to pathological lesions such as white matter lesions (WML) observed as white matter hyperintensities (WMH) on MRI. WML are thought to result from chronic hypoperfusion of the white matter (WM), which can in turn lead to oligodendrocyte damage and eventually the degeneration of their myelinating extensions [49]. Cerebral blood flow (CBF) in WM decreased close to WMH in patients with cSVD, whilst decreased CBF was also correlated with an increase in blood–brain barrier (BBB) leakage volume and rate. This correlation was strongest in regions close to WMH but was also found in the normal appearing white matter (NAWM) of these patients [65].

Increased BBB permeability and reduced CBF are hallmarks of a dysfunctional neurovascular unit (NVU), which results in a failure to provide sufficient oxygen and energy to glial cells and neurons. In the WM, oligodendrocyte and oligodendrocyte precursor cells (OPCs) are most vulnerable to the effects of hypoxia [24]. This vulnerability of OPCs to hypoxia might lead to demyelination and impairment in remyelination, leading to WML, however, how hypoxic OPCs can mediate changes in the WM, is currently not known. Previous findings show a decrease in the number of oligodendroglia after a transient period of hypoxia preceding lesion formation at the white–grey matter border [12, 29]. Demyelination was reduced or even eliminated by normalising oxygen levels in rats [10, 12]. Recent in vivo findings suggest that perivascular OPC density increases post stroke in an attempt to rescue demyelinated regions by recruiting progenitors of the damaged myelinating cells [29]. Interestingly, adult myelinating oligodendrocytes seem to be less vulnerable to hypoxia than OPC [4]. Taken together, this high vulnerability of OPC to hypoxia, particularly later stage OPC, might suggest its detrimental role in the development of WML [10, 24, 29].

It remains unknown however, if cerebral hypoperfusion occurring in cSVD patients can affect OPC and how this may lead to endothelial cell (EC) dysfunction and increased BBB permeability. The aim of this study was to investigate hypoperfusion-mediated hypoxic signalling in OPC and its impact on cerebral small vessel integrity. We hypothesised that cerebral hypoperfusion causes local hypoxia, affecting the OPC–EC signalling, leading to increased BBB permeability.

We used carotid artery stenosis as model for cerebral hypoperfusion. Although this is not an intrinsic small vessel disease model, carotid occlusive disease is associated with small vessel hypoperfusion [48]. We investigated the impact of 1 week of cerebral hypoperfusion in mice that had previously undergone bilateral carotid artery stenosis (BCAS) to identify early effects on myelin content, vascular density, BBB leakages, and hypoxic cell density. We combined our in vivo investigations with the study of OPC in vitro under hypoxic conditions. Finally, we related our findings in a retrospective analysis of VEGFA levels in blood plasma samples derived from cSVD patients.

## Materials and methods

### Animals and tissue collection

Male C57BL/6J mice were obtained from Charles River Laboratories (Sulzfeld, Germany) between 9–10 weeks of age (weighing between 20 to 26 g) and kept on a normal 12 h day-night cycle. All mice were allowed access to water and food ad libitum. All animal experiments were ethically approved by the regulatory authority of Maastricht University and were performed in compliance with the national and European guidelines (AVD1070020173885). Group sizes were calculated a priori to ensure a statistical power of 90% using G*Power 3.1 (Heinrich Heine University, Dusseldorf, Germany; www.psycholgie.hhu.de).

To mimic cerebral hypoperfusion, a hallmark for cSVD, we used the well-established BCAS mouse model. Following a period of acclimatisation, mice of 10–12 weeks old (weighting between 20 to 30 g) were randomly allocated to undergo a BCAS (*n* = 13) or a Sham surgery (*n* = 7) as described previously [56]. Briefly, mice were anaesthetised and micro coils with an internal diameter of 0.18 mm (Sawane Spring Co., Ltd, Hamamatsu, Japan) were placed around the left and right common carotid arteries in BCAS-operated mice with a 30 min delay between both sides. The same surgery was performed in Sham-operated animals without placement of the micro coils. Animals were returned to their home cages in groups of two per cage and were closely monitored daily to ensure complete recovery until they were humanely sacrificed at day 7 post-procedure. Animals were weighted before surgery and at day 1, 2, 3 and 7 after surgery. Animals received pre- and post-operative analgesia (buprenorphine 0.05 mg/kg SC pre- and post-surgery; and buprenorphine 9 µg/mL in drinking water for the first 3 days) as advised by the named veterinary surgeon.

CBF was measured during surgery by laser doppler flowmetry (LDF, moorVMS-LDF2, Moor Instruments Inc, Axminster, UK) and measurements of the cortical CBF were additionally performed under anaesthesia before (baseline), after (d0) and 7 days after (d7) surgery using a Laser Speckle Contrast Imager (LSCI, Pericam, PSI, Perimed AB, Järfälla, Sweden). Mice with a CBF reduction exceeding 50% (measured with LDF) or with a body weight reduction exceeding 15% compared to the body weight prior to surgery were humanely sacrificed and excluded from the study. Mice were sacrificed by perfusion under terminal anaesthesia 1-week post-surgery. Pimonidazole, a hypoxic marker, was injected 1 h prior to sacrifice (60 mg/kg, I.P., HypoxyProbe Inc., Burlington MA, USA) in conscious mice before CBF measurements.

Brains were harvested post-mortem following perfusion with ice-cold PBS using a peristaltic pump. A sagittal cut was used to split the brain hemispheres and the left hemisphere was fixed overnight in 4% paraformaldehyde (PFA), washed in PBS, and transferred to PBS with 0.1% sodium azide (NaN_3_) before vibratome sectioning.

### Cerebral blood flow imaging and data processing

Animals were injected with buprenorphine (0.05 mg/kg, S.C.) one hour prior to surgical procedures. Anaesthesia was initiated using isoflurane (4%) before placing mice on a stereotaxic monitoring platform (Harvard Apparatus, Hollistion, MA, USA). Heart rate, breathing rate and oxygen saturation were constantly measured during the entire procedure to monitor the animal sedation and to ensure CBF was measured in physiological conditions. Animals were kept sedated via isoflurane inhalation at a constant concentration of 1.8–2.2%. Lidocaine was injected locally onto the periosteum, followed by a midline skin incision. The skull was kept moist using a light (Cat no. 163-2129, Bio-rad laboratories, Inc., Hercules, CA, USA) and a heavy (Cat no. 330760, Sigma-Aldrich, St. Louis, MO, USA) mineral oil during imaging with the LSCI. The field of view (FOV) (1 × 1 cm) was imaged at a frame rate of 44 images/sec and resolution of 0.01 mm/pixel. Colour-coded CBF images correlated with the blood flow velocity were obtained. CBF imaging was performed before (baseline), immediately after (d0), and 7 days (d7) after surgery. At the end of d0, 6 h after the initial injection, a second injection of buprenorphine (0.05 mg/kg, S.C.) was performed, and buprenorphine administration was continued via the drinking water overnight (9 µg/mL, oral) as post-operative analgesia for all animals.

CBF images were analysed in PIMSoft (Perimed AB). CBF absolute values were obtained over the whole cortical surface (222.5 ± 1.6 mm^2^) and above the third order branch from superior sagittal sinus (8.8 ± 0.1 mm^2^) to avoid large superficial vessels. CBF values were averaged over a 1 min period. Relative CBF change was calculated as percentage of baseline measurements for both regions of interest (ROI).

CBF reduction during placement of the micro coils was monitored using LDF. After identifying the bregma and lambda, a small area was superficially thinned with a dental drill on the left primary somatosensory cortex for positioning a 0.5-mm flexible fibreoptic laser probe (Moor POF500, Moor instruments Inc.), which was removed after the surgical procedure. CBF was monitored throughout the surgery using IDEEQ (M-PAQ, Development Engineering & Evaluation [IDEE], Maastricht, The Netherlands).

### Immunohistochemistry (IHC)

Using a vibratome (VT1200S, Leica, Freiburg, Germany), 30 µm-thick coronal sections were cut, and the free-floating sections were permeabilised and blocked with 1% donkey serum in TBS-T (0.1–1% Triton-X). Sections were then incubated overnight with primary antibodies in blocking buffer. Sections were then incubated with secondary antibodies at RT for 2 h. Antibodies used are provided in Additional file 1: Table S1. Finally, the sections were mounted on gelatin-coated microscopic slides with a fluorescence-preserving mounting medium (Prolong gold antifade, Cat no. P36934, ThermoFisher Scientific).

### IHC image acquisition and analysis

Images were acquired with the experimenter masked to the group conditions using a confocal microscopy (DMI 4000 B, TCS SPE, Leica, Amsterdam, The Netherlands) or fluorescent slide scanner (ImageXpress Pico Automated Cell Imaging System, Molecular Devices, San Jose, CA, USA). To assess myelin integrity and the total number of hypoxic cells (Pimonidazole^+^), hypoxic OPC (Pimonidazole^+^/Olig2^+^/CC1^−^), nine FOVs of the corpus callosum (CC) (three per coronal section, with three sections [between + 1.3 mm and − 0.5 mm bregma]) were obtained per animal. Twenty microns thick image stacks were obtained using the confocal microscope, and processed and analysed using Fiji package [55] in ImageJ software (National Institutes of Health, Bethesda, MD, USA). The total number of hypoxic cells and hypoxic OPC were quantified in the deep cortical regions by a blinded observer and expressed in number of cells per mm^2^. Myelin content and integrity were assessed within the CC. MBP grey value signal and negative areas were measured in the CC. Early myelin damage was analysed using the anti-MBP clone SMI94 to assess focal myelin degradation [7]. Hyperintense SMI94 foci in the CC were automatically detected on maximally projected volumes using the local maxima value, after thresholding. Images for Lectin/IgG were obtained at 20×  magnification using the fluorescent slide scanner. BBB permeability was investigated in six sections (between + 2 mm and − 1 mm bregma). Sections were investigated for vascular density and extravascular leaked IgG was quantified using AngioTool [77] and ImageJ, respectively. Leakage size was measured by quantifying the area of IgG signal outside of the vascular mask delineated by the lectin signal using the Otsu threshold method. The reported total leakage size per animal corresponds to the sum of the sizes of individual leakages measured in six sections per animal (mm^2^).

### Cell cultures

Primary OPC cultures were prepared as previously described [47, 54, 58]. Briefly, cerebral cortices from new-born (P0) C57BL/6J mice were isolated, dissected and digested. Mixed glia cultures were maintained in DMEM with high glucose (Cat no. D6429, Merck Millipore, Burlington, MA, USA) containing 10% fetal bovine serum (FBS) and 1% penicillin/streptomycin (P/S) at 37 °C and 8.5% CO_2_ on poly-L-lysine (PLL) coated flasks. Medium was changed every 3–4 days and insulin (5 µg/ml) was added from day 7 onwards. Cultures were maintained for 12–14 days before initial shaking on an orbital shaker (75 rpm) at 37 °C for 45 min to remove microglia. The medium was then discarded, and flasks were shaken (280 rpm) with fresh medium overnight (16–18 h) at 37 °C. Medium was collected and plated on noncoated tissue culture dishes for 30 min at 37 °C and 8.5% CO_2_. The non-adherent cells (OPC) were collected and maintained in DMEM with high glucose containing 0.5% FBS, 1% P/S, 1% B27 supplement, bovine serum albumin (102 ng/ml), putrescine dihydrochloride (29.4 ng/ml), triiodothyronine (0.414 ng/ml), l-thyroxine (0.40 ng/ml), sodium selenite (5.00 pg/ml), progesterone (60.0 pg/ml), apo-transferrin (50 µg/ml), insulin (5 µg/ml) on PLL-coated plates. PDGF-AA (10 ng/ml), and FGF-2 (10 ng/ml) was added for 24 and 48 h, respectively, to limit spontaneous OPC differentiation before experiments (Additional file 1: Fig. S4).

The immortalised OPC line, Oli-neu (RRID:CVCL_IZ82), originally obtained by transfecting primary mouse OPC with the replication defective retrovirus expressing the t-neu oncogene [27], were maintained in DMEM with high glucose containing 10% FBS and 1% P/S at 37 °C and in an atmosphere of 5% CO_2_.

### Hypoxic primary OPC experiments

Primary OPC cultures were exposed to either normoxic (21% oxygen) or hypoxic (2% oxygen) conditions for 24 h at 37 °C and 8.5% CO_2_ in fresh PDGF-AA and FGF-AA free OPC medium. After 24 h, medium was collected, and cells were lysed and stored at − 20 °C before RNA isolation.

### RNA isolation and sequencing

Primary OPC were lysed using RLT buffer (Qiagen, Hilden, Germany) and frozen at − 80 °C. Samples were later thawed, and RNA was isolated using the RNeasy Micro kit following the manufacturer’s instructions (Cat no. 74004, Qiagen). RNA quantity was checked using Qubit 2.0 Fluorometer (Invitrogen, Waltham, MA, USA) and RNA quality was assessed using Bioanalyzer (Cat no. RNA 6000 Nano kit; 2100 Bioanalyzer, Agilent Technologies, Santa Clara, CA, USA). Purification of mRNA from total RNA (NEXTFLEX Poly(A) Beads 2.0, Cat no. NOVA-512992, PerkinElmer, Waltham, MA, USA) and directional, strand specific RNA library preparation (NEXTFLEX Rapid Directional RNA-Seq Kit 2.0, Cat no. NOVA-5198, PerkinElmer) was performed according to manufacturer’s protocol. Sequencing was performed using NovaSeq 6000 Sequencing system (NovaSeq S Prime flow cell 200 cycles; NovaSeq 6000, Illumina, Inc, San Diego, CA, USA) according to manufacturer’s protocol.

### RNA sequencing analysis

The raw sequencing data was trimmed using fastp. The remaining reads were mapped against the Ensembl mouse genome (release 100) using STAR (version 2.7.3a) and quantified using RSEM (v.1.3.1). The resulting raw read counts were processed using the R package DESeq2. Genes that were not sequenced (0 reads) in more than 75% of the samples of any given condition were removed. Genes were considered differentially expressed with an adjusted p-value (false discovery rate; FDR) below 0.01. Kyoto Encyclopedia of Genes and Genomes (KEGG) enriched pathways, Gene ontology (GO) classification and UniProt functional annotation, terms approximating cellular component, biological process, and molecular function, were used to identify functional enriched differentially expressed genes (DEG) using the Database for Annotation, Visualization, and Integrated Discovery (DAVID) v6.8 [22, 23]. The modified Fisher exact p-value (EASE score) < 0.05 and FDR < 0.05 were considered enriched.

### Hypoxic Oli-neu experiments

The immortalized OPC cell line, Oli-neu, was exposed to either normoxic or hypoxic conditions (as above) for 12, 24, or 48 h at 37 °C and 5% CO_2_. Medium was collected, and cells were lysed for RNA isolation and stored at − 20 °C. Experiments were repeated in cells transfected with Negative control siRNA (Silencer™ Cy™3-labeled Negative Control No. 1 siRNA, Cat no. AM4621, Invitrogen), *Hif1α* siRNA (Silencer™ Select Pre-Designed mouse *Hif1α* siRNA, sequence 5′ → 3′: Sense CCUUUACCUUCAUCGGGAAAtt; Antisense UUUCCGAUGAAGGUAAAGGag, Cat no. 4390771, Invitrogen) and/or *Epas1* siRNA (Silencer™ Select Pre-Designed mouse Epas1 siRNA, sequence 5′ → 3′: Sense CGGAUCCACCAUUACAUUUtt; Antisense AAAUGUAAUGGUGGAUCCGgg, Cat no. 4390771, Invitrogen). Briefly, cells were transfected with 20 pmol siRNA and 6 µg/ml Lipofectamine™ 2000 Transfection Reagent (Cat no. 11668, Invitrogen) in high glucose DMEM containing 10% FBS and 10% Opti-MEM (Cat no. 31985070, ThermoFisher Scientific) for 24 h. Cells were then washed with PBS and incubated at normoxic or hypoxic conditions with normal culturing medium. After 24 h, cells were lysed and stored at − 20 °C until further use.

### Conditioned medium experiments

Immortalised mouse brain EC (bEnd.3) cells were cultured with culture medium diluted (1:1) with conditioned medium (CM) obtained from Oli-neu exposed to normoxic or hypoxic conditions for 24 h. After 24 h, cells were lysed for RNA isolation and stored at − 20 °C until further use.

### Quantitative PCR

Total RNA was isolated from Oli-neu cells using TRIzol Reagent (Invitrogen) according to the manufacturer’s protocol and stored at − 80 °C before use. Quality and quantity were checked using NanoDrop 1000 spectrophotometer and the RNA was reverse transcribed into cDNA using the high-capacity RNA-to-cDNA kit (Cat no. 1708891, Bio-rad laboratories, Inc.) according to manufacturer’s manual. cDNA samples were stored at − 20 °C before use. Quantitative PCR was performed using Sensimix™ SYBER® & Fluorescein kit (Cat no. QT615-05, Meridian Bioscience Inc., Cincinnati, OH, USA) on the Light Cycler 480 (Roche Applied Science, Penzberg, Germany) with the following qPCR program: 10 min at 95 °C followed by 55 cycles a 10 s at 95 °C and 20 s at 60 °C. Temperature was increased from 60 to 95 °C for melting curve analyses. Primers were designed to cover exon-exon junctions and all possible splice variants using NCBI Primer-BLAST tool. Primers were synthesised by Eurofins Genomics (Ebersberg, Germany) and quality was ensured by testing on appropriate tissue or cell cultures, as well as by calculation of primer efficiency employing a cDNA dilution curve. Two stable reference housekeeping genes (Rpl13a and Ywhaz) were selected from a selection of three genes by using the GeNorm Software (Primerdesign, Southampton, NY, USA). Primers are listed in Additional file 1: Table S2. Gene expression analysis was performed using LinReg PCR (Ver. 2014.0) and the Light Cycler 480 data converter (Ver. 2014.1).

### Enzyme-linked immunosorbent assay (ELISA)

CM was obtained from primary OPC or Oli-neu cells after exposure to either 21% or 2% O_2_ for 24 h. Secreted VEGFA concentration was determined in diluted (1:1) samples using commercially available Mouse VEGF-A ELISA kit (Cat no. BMS619-2, Invitrogen) according to manufacturer’s protocol.

### Human brain imaging analysis

#### Study population

We performed a retrospective pilot study, for which we used data of an MRI study in cSVD. Eighty patients with clinically manifested cSVD (lacunar stroke or vascular cognitive impairment) and 39 age- and sex- matched controls were included. Inclusion criteria, and methods for image acquisition, processing and analysis have been described previously [28, 64, 65, 72].

The Medical Ethics Committee of the Maastricht University Medical Centre approved the study. All participants were included after providing written informed consent according to the Declaration of Helsinki. This study is registered on trialregister.nl (NTR number: NTR3786). Participants were included from the Maastricht University Medical Centre and Zuyderland Hospital, the Netherlands, between April 2013 and December 2014.

#### Structural MRI

All participants underwent structural brain imaging on a 3.0T MRI system (Achieva TX, Philips Healthcare, Best, the Netherlands) using a 32-element head coil suitable for parallel imaging. T1-weighted sequence was used for anatomical reference and T2-weighted fluid-attenuated inversion recovery (FLAIR) sequence for detecting WMH [28, 72].

#### Permeability MRI

Details of the scanning protocol have been published [72]. Dual-time resolution dynamic contrast enhanced (DCE)-MRI consisted of a fast and slow dynamic scan time sequence to sample properly the various time-scales in the contrast enhancement curves. Scans of both sequences were acquired prior to contrast injection, followed by the fast sequence during contrast bolus injection (Gadobutrol), and then followed by the slow sequence.

#### Image processing and analysis

##### Tissue segmentation

Grey and white matter were segmented on T1-weighted images using Freesurfer [15]. NAWM and WMH were differentiated by segmenting WMH on FLAIR images automatically, with manual corrections by a trained assessor [11]. T1 and FLAIR Images were coregistered using FSL (v5.0) and the NAWM and WMH were selected [26].

##### Pharmacokinetic modelling

DCE-MRI data was pharmacokinetically modelled and histogram analysis was performed as previously described [17, 73]. Briefly, contrast agent tissue concentration was calculated by using the relative signal change over time and T1 mapping, using the superior sagittal sinus as vascular input function [33]. Subsequently, the Patlak pharmacokinetic model was used to calculate the BBB leakage rate in terms of the leakage rate K_i_ (min^−1^) [50].

##### Histogram analysis

K_i_ was determined in a voxel wise manner [28, 65, 72]. For both NAWM and WMH, a K_i_ voxel histogram was composed, and noise correction was applied by mirroring and then subtracting negative K_i_ value distribution from the original K_i_ distribution, resulting in a histogram of K_i_ values reflecting detectable leakage rates. Mean leakage rate (mean K_i_) was calculated by taking the average of all noise-corrected K_i_ values. The relative volume of leakage with respect to the tissue volume, the fractional leakage volume (v_L_), was the remaining area under the noise corrected histogram curve [72].

#### Retrospective blood plasma VEGFA analysis

Blood samples from all participants were collected. Plasma supernatant was obtained from these samples and stored for future analysis at − 80 °C. Plasma VEGFA levels were determined with the Human VEGF-A ELISA kit (Cat no. BMS277-2, Invitrogen) according to manufacturer’s protocol. Samples with absorption values below blank measurements were considered artefacts and excluded from further analysis.

### Statistical analysis

Data were analysed in GraphPad Prism 9 (Dotmatics) or SPSS version 28 (IBM Corp., San Diego, CA, USA). Unpaired Student t-tests or Mann–Whitney tests (for non-parametric data) were used to compare Sham vs BCAS, normoxia vs hypoxia exposed cells, and VEGFA in controls vs cSVD patients. One-way ANOVA with post-hoc Tukey’s multiple comparisons tests was used to assess multiple comparisons. Uni- and multivariable regression analysis was performed with group (cSVD or control) as independent and MRI measures (WMH volume, and BBB leakage K_i_ and v_L_ in NAWM and in WMH) as dependent variables, and correction for age and sex. Outliers for VEGFA plasma concentration were identified (defined as 3rd quartile + 1.5*interquartile range). The relation between VEGFA plasma concentration and MRI characteristics was assessed with uni- and multivariable regression analysis, with correction for age and sex, for both patients and controls after removal of outliers (details on outliers are provided in Additional file 1: Table S4). Analyses without exclusion of outliers are provided in the supplementary data. Estimated 95% confidence interval were obtained with standardised Z-scores. *P* < 0.05 was considered statistically significant and data are expressed as mean ± SEM.

## Results

### BCAS led to a persistent cerebral blood flow reduction after 7 days

**Fig. 1:**
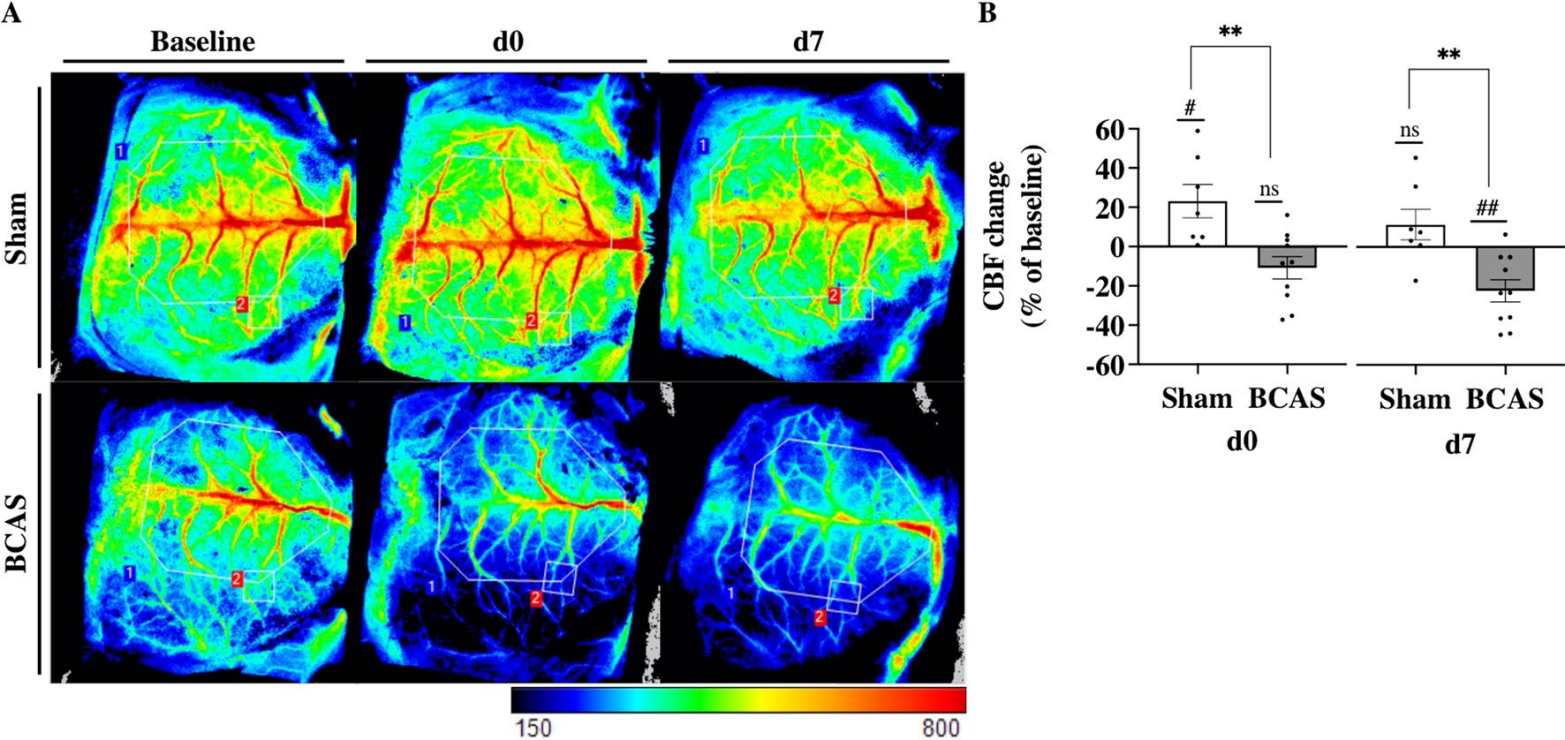
Bilateral carotid artery stenosis led to a persistent decrease in cerebral blood flow. (**A**) LSCI images showing blood flow in superficial blood vessels captured at baseline, d0, and d7. Blood flow measurements in ROI1 (blue) contained all sizes superficial blood vessels, while ROI2 (red) contained the 3^rd^ order branch from superior sagittal sinus to avoid larger superficial vessels. Visualization of blood flow signal ranged from high flow (red) to low flow (blue). (**B**) Changes in CBF were quantified and compared to baseline measurements at d0 and d7 (ROI2). Scale bar, arbitrary value, 150 (low blood flow), 800 (high blood flow). Mean ± SEM; ns = not significant; #*p* < 0.05, ##*p* < 0.01 vs baseline measurements; **p < 0.01, vs Sham; unpaired student t-test

Three mice in the BCAS group had to be sacrificed due to post-operative complications, whereas full recovery was seen in the rest (Sham: *n* = 7; BCAS: *n* = 10; Additional file 1: Fig. S1). Blood flow was measured in a ROI containing all sizes of superficial vessels (ROI1, Fig. 1A), and a smaller ROI above the 3rd order branch from superior sagittal sinus to avoid larger superficial vessels (ROI2, Fig. 1A). A significant decrease in CBF in ROI1 was observed in BCAS mice compared to baseline measurements at d0 (− 14.7 ± 5.3%, *p* = 0.02) and at d7(− 27.2 ± 3.6%, *p* < 0.0001), while no significant changes were observed in Sham mice (Additional file 1: Fig. S1E). CBF differed between BCAS and Sham at both d0 (*p* = 0.013) and d7 (*p* < 0.0001) (Additional file 1: Fig. S1E). At d0, CBF in ROI2 was not significantly decreased in BCAS mice (− 10.7 ± 5.7%, *p* = 0.089) compared to baseline measurements, while CBF was increased in Sham mice (+ 23.2 ± 8.5%, *p* = 0.034, Fig. 1B). At d7, CBF in ROI2 was significantly decreased in BCAS mice (− 22.3 ± 5.6%, *p* < 0.01) compared to baseline, while CBF was normalised in Sham mice (+ 11.4 ± 7.8%, Fig. 1B). CBF in ROI2 differed between BCAS and Sham at both d0 and d7 (*p* < 0.01).

### Cerebral hypoperfusion did not alter myelin integrity after 7 days

**Fig. 2:**
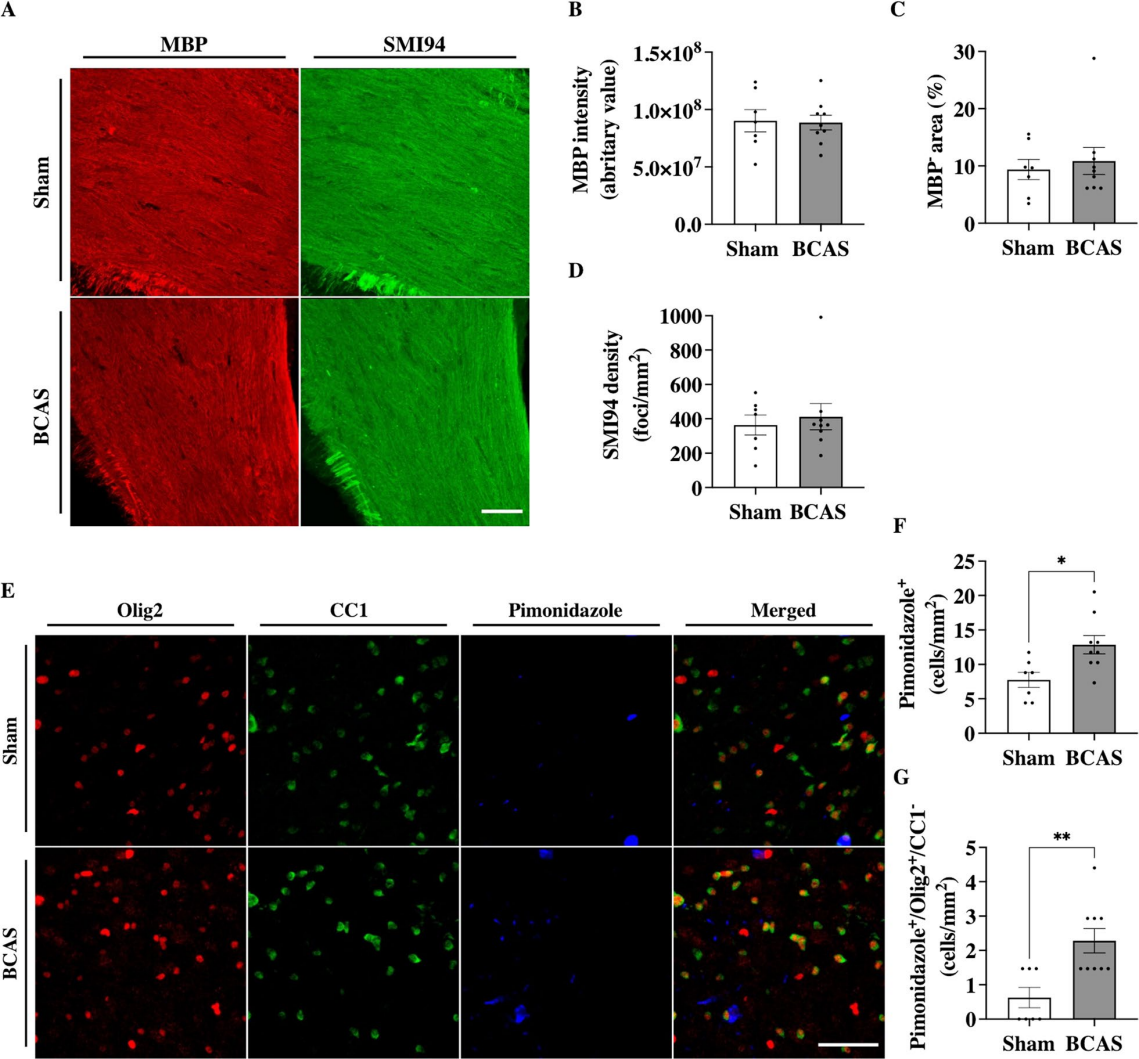
BCAS led to an increase in hypoxic OPC in deep cortical regions after 7 days. (**A**) Immunolabeling for MBP and MBP clone SMI94 in the corpus callosum. Scale bar, 50 µm. (**B**) Quantification of MBP integrity, (**C**) MBP negative area, and (**D**) Myelin degradation, quantified by intensity signal and the number of hyperintense foci, respectively, was not statistically different between BCAS and Sham mice 7 post-operative days. (**E**) immunolabeling for Olig2, CC1, and Pimonidazole-Pacific blue in the deep cortical regions. Scale bar, 50 µm. (**F**) A significant increased hypoxic cell density was observed in the deep cortical regions of BCAS animals compared to Sham animals. (**G**) BCAS mice also showed an increased number of hypoxic OPC in these regions compared to Sham animals. Mean ± SEM; *p < 0.05, **p < 0.01; unpaired student t-test or Mann–Whitney U-test

Structural changes in the CC were investigated ex vivo by an immunostaining for MBP (Fig. 2A). MBP intensity and MBP negative area analysis showed no significant structural changes in the CC BCAS compared to Sham mice (*p* = 0.93 and *p* = 0.90, respectively, Fig. 2B, C). SMI94 was used to investigate early myelin changes. SMI94 detects an MBP peptide known to only be exposed during myelin degradation [39]. There was no difference in the total number of hyperintense foci between BCAS and Sham mice (*p* = 0.65, Fig. 2D).

### Cerebral hypoperfusion led to increased hypoxia in OPCs in the deep cortical regions

Immunolabeling for Olig2, CC1, and pimonidazole revealed OPCs, oligodendrocytes, and hypoxic cell densities in the deep cortical regions, respectively (Fig. 2E). A significant increase in hypoxic cells was observed in BCAS mice when compared to Sham (12.9 ± 1.3 vs 7.8 ± 1.1 cells/mm^2^; p = 0.013, Fig. 2F). Olig2 and CC1 signals in hypoxic cells were thereafter used to identify hypoxic OPCs and oligodendrocytes. Hypoxic CC1 cells were not detected. In contrast, hypoxic Olig2 cells were increased in abundance in the deep cortical regions of BCAS mice compared to Sham (2.3 ± 0.4 vs 0.6 ± 0.3 cells/mm^2^, p = 0.004, Fig. 2G). Neither OPCs nor oligodendrocyte density was altered in BCAS mice compared to Sham (Additional file 1: Fig. S3).

### Hypoxia in OPC led to increased VEGFA secretion in vitro

**Fig. 3:**
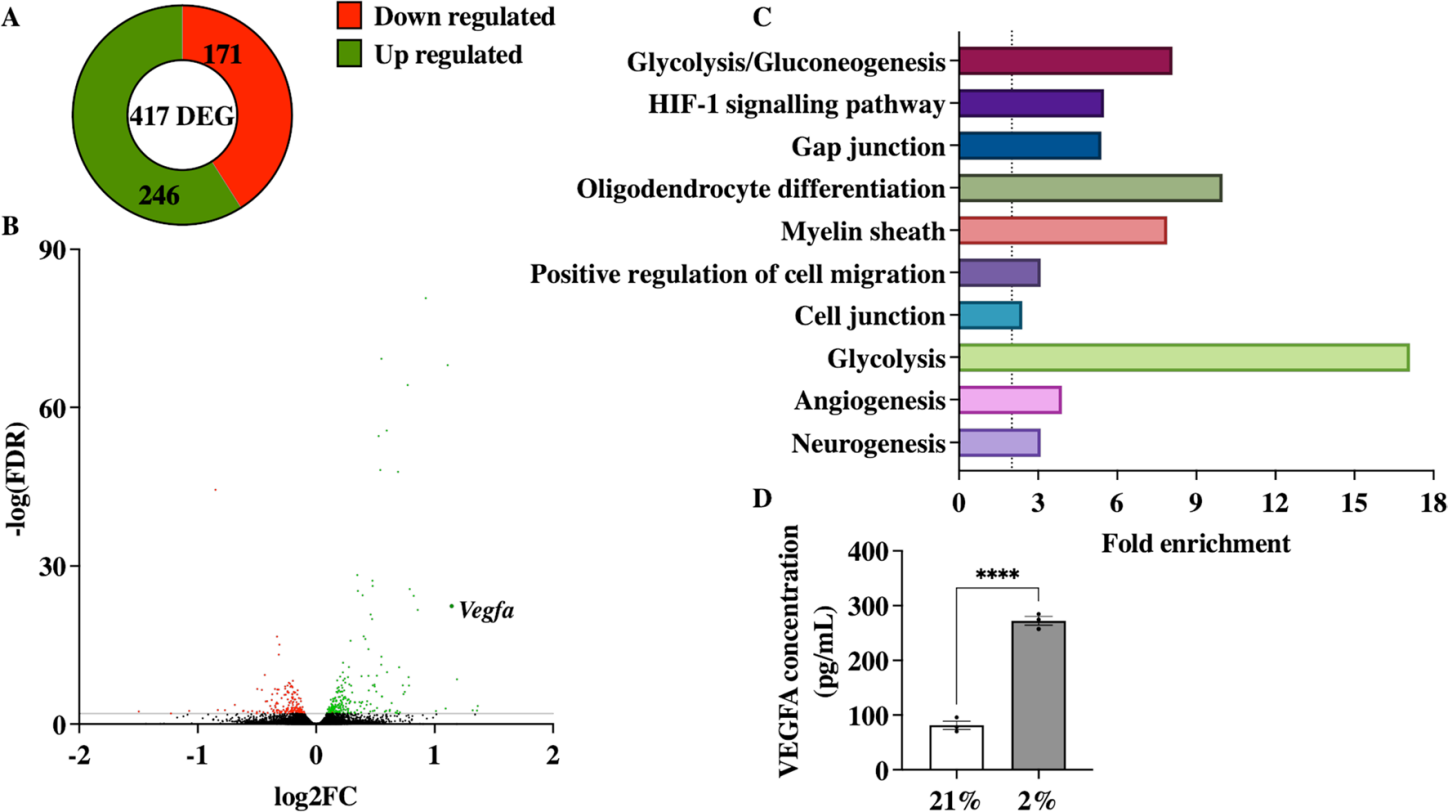
Hypoxia induced the expression of 417 DEGs. (**A**) Hypoxia (2% O_2_) induced the expression of 417 differentially expressed genes (DEG) compared to normoxia (21% O_2_) in OPC, of which 256 were upregulated and 171 downregulated. (**B**) Volcano plot showing the 417 DEG in hypoxic OPC with genes that were downregulated in red, and upregulated genes in green (**C**) DAVID enrichment analysis shows enrichment of pathways including HIF-1 signalling pathway, positive regulation of cell migration, and angiogenesis. Terms were considered significant with an EASE score < 0.05 and FDR < 0.05. (**D**) VEGFA was identified as a potential key mediator in these pathways and secreted protein concentration in the hypoxia exposed conditioned medium was increased compared to normoxia. Mean ± SEM; ****p < 0.0001, vs 21%; unpaired student t-test

To further investigate the effects of hypoxia in OPC, we cultured primary OPC in hypoxic condition. We then identified DEG in normoxic vs hypoxic OPC by RNA sequencing. This led to the identification of 417 DEG with an FDR below 0.01, of which 246 were up-regulated and 171 down-regulated (Fig. 3A). Figure 3B represents the volcano plot, with all upregulated DEGs in green and the down regulated DEGs in red. DAVID enrichment analysis was performed for the functional annotation of the identified DEG (Fig. 3C). HIF-1 signalling pathway (5.5-fold change [FC], FDR < 0.0001), positive regulation of cell migration (3.1 FC, FDR = 0.047), cell junction (2.4 FC, FDR < 0.0001), and angiogenesis (3.9 FC, FDR = 0.014) were considered as enriched pathways that might be involved in crosstalk of OPC with surrounding cells. Vascular endothelial growth factor A (*Vegfa*) was identified as a potential mediator in the mentioned enriched pathways (log2FC = 1.14; − log(FDR) = 22.4).

To investigate the potential of VEGFA protein as mediator of crosstalk, secreted protein levels in the CM of these cells were measured. Hypoxia exposure led to a significant increase in VEGFA protein secretion by primary OPCs compared to normoxic condition (272.2 ± 7.9 vs 81.6 ± 7.5 pg/ml, *p* < 0.0001; Fig. 3D).

### VEGFA increase in hypoxic OPCs was mediated by *Hif1α *and *Epas1*

**Fig. 4:**
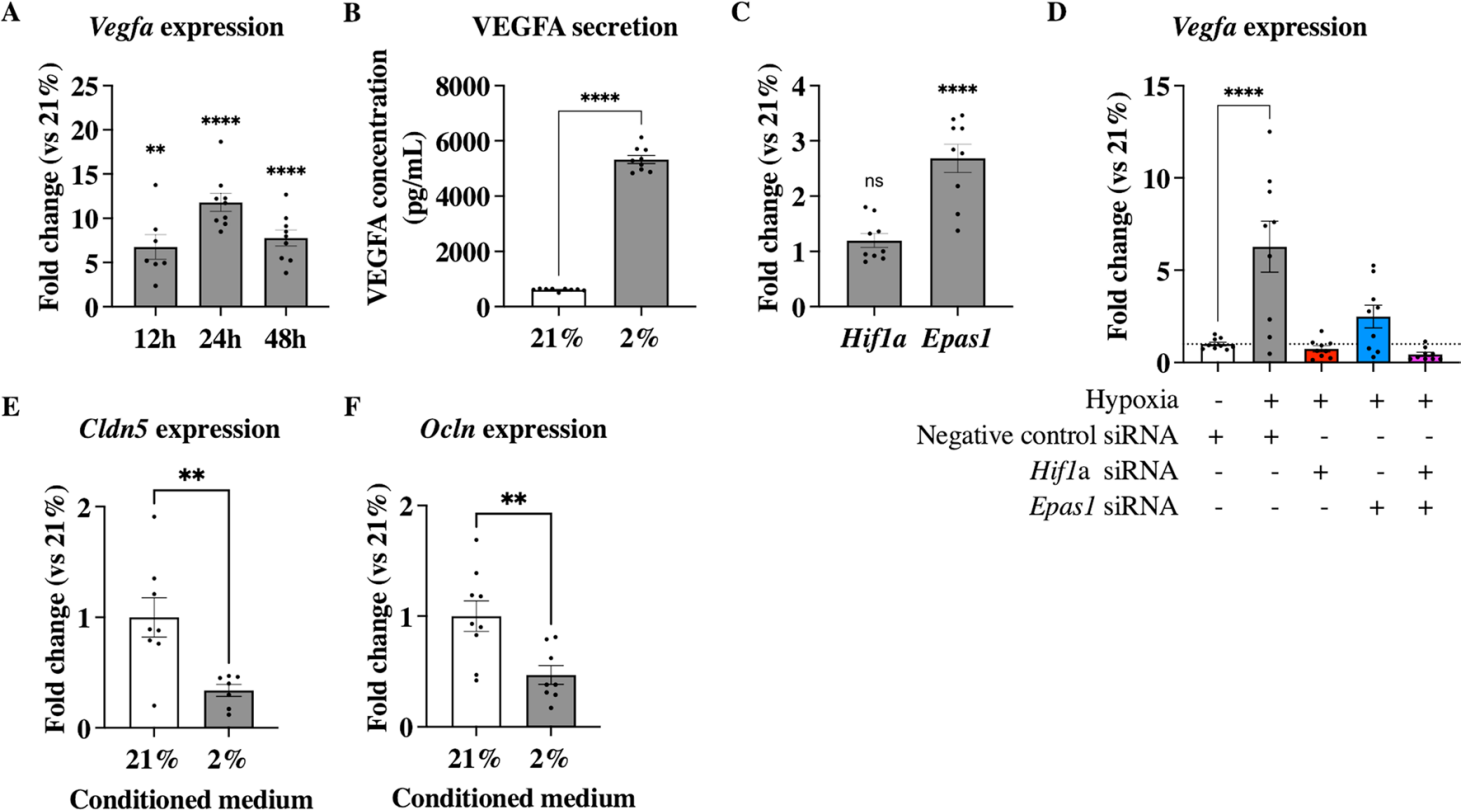
Hypoxia induced expression and secretion of VEGFA in Oli-neu cells. (**A**) The expression of *Vegfa* mRNA was constantly increased in hypoxic Oli-neu cells compared to normoxic cell expression from 12 to 48 h, with a peak increase after 24 h. (**B**) Hypoxic Oli-neu cells significantly secreted more VEGFA protein into conditioned media 24 h after exposure to hypoxia compared to normoxia. (**C**) Oli-neu cells exposed to hypoxia did not significantly increase mRNA expression of *Hif1α* after 24 h exposure, while *Epas1* (aka *Hif2α*) was significantly increased compared to normoxic cell expression. (**D**) inhibition of *Hif1α* and/or *Epas1* results in inhibition of hypoxia mediated increase in *Vegfa* expression. (**E**) Both *Claudin-5* (*Cldn5*) and (**F**) *Occludin* (*Ocln*) mRNA expression was significantly decreased in brain EC treated with hypoxic OPC derived CM. Mean ± SEM; ns = not significant, **p < 0.01, ****p < 0.0001, vs 21%; unpaired student t-test or one-way ANOVA with Tukey’s multiple comparisons test

Expression of *Vegfa* mRNA was measured in OPCs after 12 h, 24 h, and 48 h hypoxia exposure and showed a persistent significant increase in mRNA expression (12 h: 6.75 ± 1.40 FC, *p* < 0.01; 24 h: 11.81 ± 1.02FC, *p* < 0.0001; 48 h: 7.78 ± 0.91 FC,* p* < 0.0001; Fig. 4A). This increase in *Vegfa* expression translated in secreted VEGFA protein concentration as measured in hypoxia exposed CM at 24 h compared to normoxia (5329 ± 145.4 vs 617.8 ± 15.83 pg/ml, *p* < 0.0001; Fig. 4B). Expression of *Hif1α*, the most important gene involved in hypoxic signalling, was not significantly increased in hypoxia exposed OPC compared to normoxic cells (1.20 ± 0.13 FC; Fig. 4C), while expression of *Epas1*, one of the DEGs involved in angiogenesis and related to hypoxia and VEGFA signalling [36]*,* was significant increased (2.68 ± 0.26 FC, *p* < 0.0001; Fig. 4C). OPC transfected with negative control siRNA and exposed to hypoxia showed a significant increase of *Vegfa* expression (6.28 ± 1.38FC, *p* < 0.0001; Fig. 4D) compared to normoxia, while inhibition of *Hif1α* resulted in normalisation of *Vegfa* expression (0.75 ± 0.16 FC compared to normoxia, *p* = 0.99; Fig. 4D). *Epas1* inhibition decreased *Vegfa* in hypoxic cells significantly and was thus not significantly different from normoxic expression (2.49 ± 0.62 FC, *p* = 0.54, Fig. 4D). Inhibition of expression of both genes decreased *Vegfa* expression by twofold compared to normoxia (0.44 ± 0.11 FC, *p* = 0.98, Fig. 4D).

As VEGFA is known to regulate the expression of tight junction proteins, Claudin-5 (*Cldn5*) and Occludin (*Ocln*) mRNA expressions were measured in mouse bEnd.3 ECs treated with CM from either normoxic or hypoxic Oli-neu cells [2, 3, 32, 44, 61]. Both *Cldn5* and *Ocln* expression in bEnd.3 cells were significantly decreased when treated with hypoxic CM (0.34 ± 0.05 FC, *p* = 0.005 and 0.47 ± 0.09 FC,* p* = 0.006, respectively; Fig. 4E, F).

### Hypoperfusion led to increased BBB permeability in mice after 7 days

**Fig. 5:**
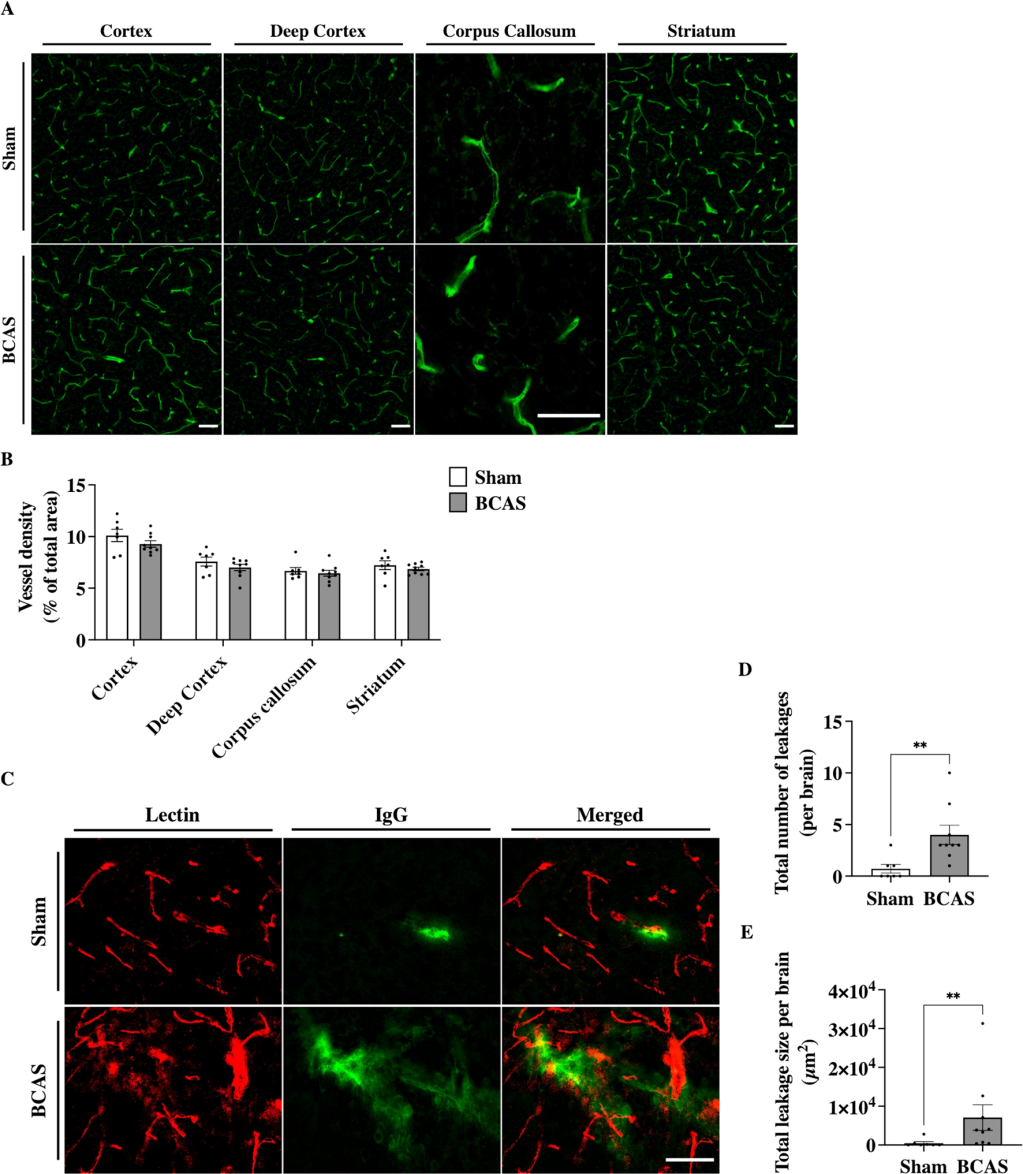
BCAS led to an increase in BBB permeability after 7 days without changes in vascular density. (**A**) immunolabeling for lectin in the cortex, deep cortex, corpus callosum, and striatum. Scale bar, 50 µm. (**B**) Quantification of vessel density in BCAS compared to Sham. No statistical differences were found in vessel density when comparing BCAS to Sham in the respective areas. (**C**) Immunolabeling for blood vessel (Lectin), and mouse IgG for BBB leakages. Scale bar, 50 µm. (**D**) The number of leakages and (**E**) the total leakage size was significantly higher in BCAS compared to Sham. Mean ± SEM; **p < 0.01, vs Sham; unpaired student t-test

The effects of VEGFA play a crucial role in the regulation of angiogenesis and BBB permeability [40, 70]. Thus, we investigated vascular density and BBB permeability changes in our BCAS mice. After 1 week of hypoperfusion, vascular density was not changed in the cortex (*p* = 0.22), deep cortex (*p* = 0.28), CC (*p* = 0.60), or striatum (*p* = 0.39) when comparing BCAS to Sham (Fig. 5A, B). We then assessed BBB permeability by quantifying IgG extravasation in BCAS and Sham mice (Fig. 5C). The number of leakages in BCAS mice was significantly higher compared to Sham (4.00 ± 0.93 vs 0.71 ± 0.42 leakages/brain, *p* < 0.01, Fig. 5D). Furthermore, the total leakage size was significantly higher in BCAS compared to Sham (7079 ± 3297 vs 480 ± 390 mm^2^, *p* < 0.01, Fig. 5E).

### Increased VEGFA plasma levels in cSVD patients correlated with increased BBB permeability in NAWM

We excluded samples considered as artefacts and 12 samples showing outliers for VEGFA concentration (eight patients and four controls) from further analysis. This resulted in 47 samples of cSVD patients and 26 controls; characteristics are presented in Table 1.

**Table 1:** Characteristics of the clinical study population

		Controls (n = 26)	cSVD Patients (n = 47)	Univariable	Multivariable (age and sex corrected)
				β (p-value)	β (p-value)
	Age (years)	67.8 ± 2.5	69.6 ± 1.6		
	Male (Female)	18 (8)	30 (17)		
WMH	Relative volume, 10^–3^	3.5 ± 1.8	16.1 ± 2.4	0.390 (< 0.001)	0.412 (< 0.001)
BBB leakage in NAWM	K_i_, 10^−3^ min^−1^	1.09 ± 0.07	0.98 ± 0.05	− 0.176 (0.137)	− 0.182 (0.129)
	v_L_	0.276 ± 0.034	0.370 ± 0.028	0.241 (0.040)	0.253 (0.035)
BBB leakage in WMH	K_i_, 10^−3^ min^−1^	0.85 ± 0.06	0.84 ± 0.04	− 0.016 (0.893)	− 0.033 (0.723)
	v_L_	0.318 ± 0.037	0.459 ± 0.031	0.318 (0.006)	0.328 (0.005)

Characteristics in mean ± SEM unless otherwise indicated. Association between WMH, BBB leakage rate (K_i_) and volume (V_L_) and participant group was determined using a uni- and multivariable (corrected for age and sex) analysis. Standardised Coefficients Beta (β) and p-value are shown for each analysis. P < 0.05 was considered as significant

*WMH* white matter hyperintensities, *BBB* blood–brain barrier, *NAWM* normal appearing white matter, *K*_*i*_ leakage rate, *v*_*L*_ leakage volume

**Fig. 6:**
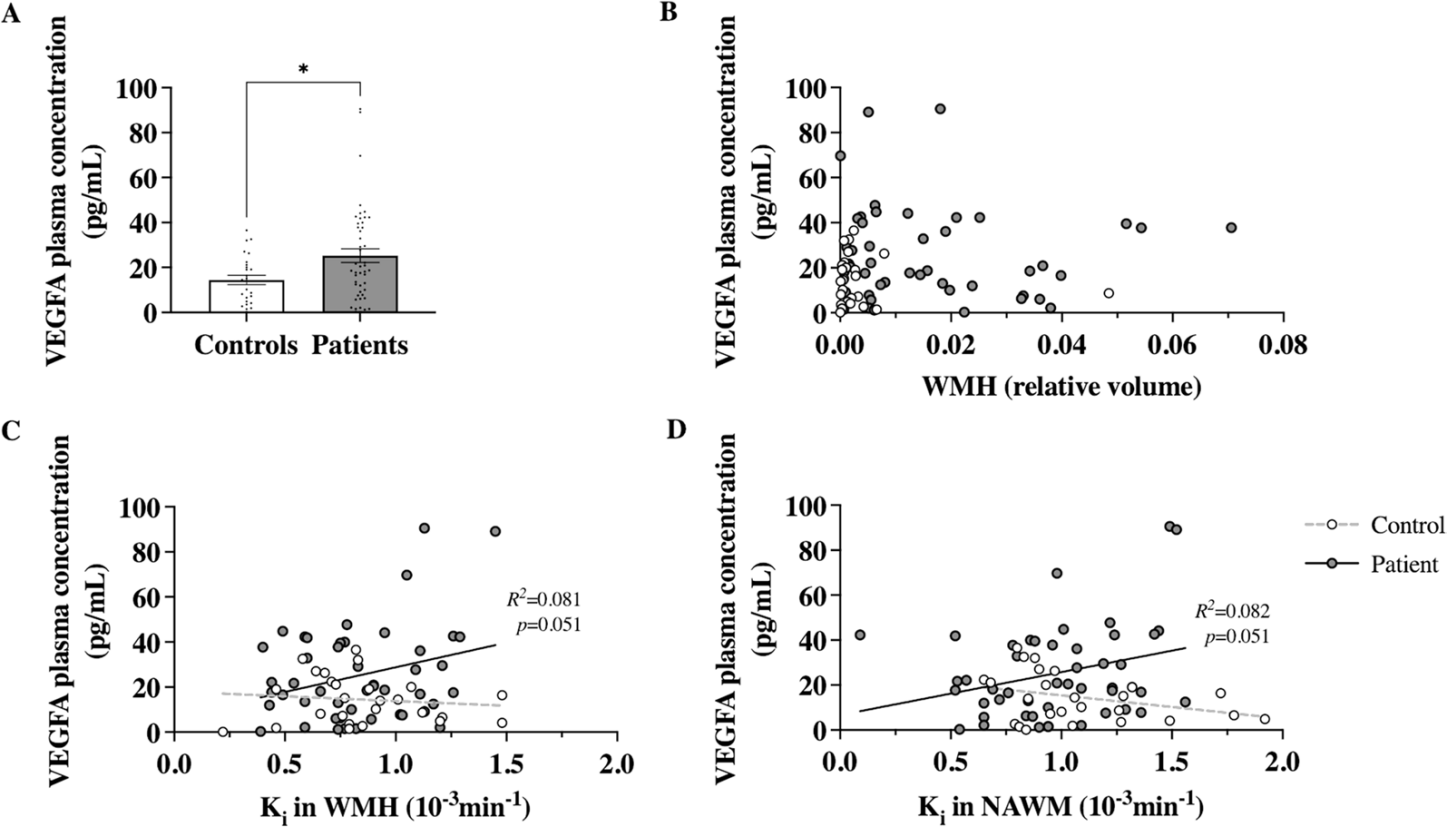
Increased VEGFA plasma levels in cSVD patients. (**A**) VEGFA blood plasma levels in cSVD patients were higher compared to age and sex-matched controls. (**B**) There was no correlation between VEGFA plasma levels and WMH volume in patients or controls. A trend in the relation between VEGFA plasma levels and leakage rate in (**C**) WMH and (**D**) NAWM in patients, but not in controls, was observed (indicated by the solid for patients and dotted line for controls). Abbreviations: WMH = white matter hyperintensities; NAWM = normal appearing white matter; K_i_ = leakage rate. For quantification, mean ± SEM; *p < 0.05; Mann–Whitney U-test

Higher WMH volume, and higher BBB leakage volumes (V_L_) both in NAWM and WMH were significantly associated with cSVD patient group (Table 1). Plasma VEGFA concentration was higher in cSVD patients compared to controls (25.26 ± 3.07 vs 14.45 ± 2.08 pg/ml,* p* = 0.035, Fig. 6A). In cSVD patients, we found a trend in the relation between VEGFA plasma concentration and BBB leakage rates K_i_ in the NAWM and WMH (*p* = 0.051 and *p* = 0.051, respectively; Table 2 and Fig. 6C, D). However, age and sex are known to influence both VEGFA plasma levels and BBB integrity [9, 53, 62, 68]. After adjustment for these covariates we found a significant the relationship between VEGFA plasma levels and BBB leakage rate (K_i_) in the NAWM in patients (*p* = 0.044, Table 2). VEGFA plasma levels were not associated with leakage rate in the WMH, leakage volume in NAWM and WMH, or WMH volume after correcting for age and sex (Table 2). In controls, no associations were found between VEGFA plasma levels and any of the imaged variables.

**Table 2:** Association between increased VEGFA plasma levels and BBB leakage rate in the NAWM of cSVD patients

		Univariable		Multivariable (age and sex corrected)	
		β [95%CI]	*p*-value	β [95%CI]	*p*-value
WMH		− 0.024 [− 0.324, 0.276]	0.873	0.009 [− 0.277, 0.294]	0.951
BBB leakage in NAWM	K_i_	0.286 [− 0.001, 0.574]	0.051	0.307 [0.009, 0.605]	0.044
	v_L_	− 0.217 [− 0.510, 0.076]	0.142	− 0.255 [− 0.556, 0.045]	0.093
BBB leakage in WMH	K_i_	0.286 [− 0.001, 0.574]	0.051	0.284 [− 0.016, 0.585]	0.063
	v_L_	− 0.170 [− 0.466, 0.126]	0.253	− 0.208 [− 0.511, 0.095]	0.174

Univariable analysis was conducted with MRI characteristics as dependent and VEGFA as independent variable. Multivariable analysis was done by correcting for age and sex. Standardised Coefficients Beta (β) with the estimated 95% confidence interval (95%CI) and p-value are shown for each analysis. P < 0.05 was considered significant

*WMH* white matter hyperintensities, *BBB* blood–brain barrier, *NAWM* normal appearing white matter, *K*_*i*_ leakage rate, *v*_*L*_ leakage volume, *CI* confidence interval

## Discussion

The aim of this study was to investigate the impact of cerebral hypoperfusion on early mechanisms involved in the development of BBB dysfunction and WM damage. We showed that cerebral hypoperfusion in mice, induced by carotid stenosis, leads to an increase in hypoxia in OPC residing in the deep cortical regions. Additionally, an increase in BBB permeability was observed without clear changes in vascular density and WM integrity. In vitro hypoxic conditions led to increased *Hif1α-* and *Epas1*-dependent VEGFA expression and secretion in OPC, leading to inhibition of tight junction (TJ) proteins *Cldn5* and *Ocln* expression in brain EC. Finally, we showed a significant increase in plasma levels of VEGFA protein, derived from patients with intrinsic cSVD compared to age-matched controls. VEGFA levels correlated with BBB permeability in the NAWM of these patients, suggesting a potential role in the early mechanisms leading to BBB dysfunction and the development of WMH.

In our mouse experiments, CBF was decreased in BCAS at 7 days after surgery, while the CBF after Sham operation did not differ from baseline measurements. Similar results were found on CBF reduction at 2 h after BCAS and no changes in Sham-operated mice [19, 25, 31, 42, 46, 56, 57]. Taken together, we confirm a sustained decrease in CBF after 1 week in the BCAS model. To investigate the effects of hypoperfusion on myelin integrity and WML, we examined the MBP integrity in the CC of mice after 7 days of hypoperfusion. Our results showed no changes in myelin integrity, which is in line with earlier studies showing myelin damage due to cerebral hypoperfusion in BCAS mice only after 14 days, but not after 7 days [8, 37, 42, 56, 57].

Although myelin damage could not be detected after 7 days, an increased number of hypoxic cells were observed in the cortical region of BCAS mice. Previous findings also indicated a hypoperfusion-mediated increase in cerebral hypoxia and hypoxia in the CC after 21 and 28 days, respectively [13, 31]. This apparent hypoxic susceptibility was also identified in a study using magnetic resonance angiography and arterial spin labelling to measure intracranial, cortical, and subcortical CBF in BCAS mice. There was a more substantial and lasting decrease in blood flow in the subcortical areas compared to the brain surface in BCAS mice [19]. We found a substantial proportion of hypoxic cells to be OPC, while no mature oligodendrocytes were found positive for the hypoxic marker. Cerebral hypoperfusion is known to induce OPC proliferation and increase OPC density at 14 postoperative days in mice, and a decrease in mature oligodendrocytes after 4 weeks [16, 37]. We did not observe any changes in OPC density after 7 days. These results suggest that the effects of hypoperfusion mediated hypoxia on OPC might precede the effects on OPC maturation and myelin integrity.

When exposing OPC to hypoxia in vitro*,* pathway enrichment analysis of our transcriptomic data revealed the regulation of oligodendrocyte differentiation and HIF-1 signalling as well as angiogenesis, highlighting a possible interaction between hypoxic OPC and EC. This unbiased approach helped us identify VEGFA as a potential candidate in this interaction. Of note, previous studies have suggested the regulation of other factors, such as *Wnt7a/b*, *Sox9/10*, or several matrix metalloproteinases (*MMPs*), in hypoxic OPC [1, 18, 41, 42, 69, 75]. However, we did not find these factors to be regulated in our transcriptomic data derived from hypoxic OPC. *Sox10* was differentially expressed in our RNA data but was not involved in differentiation, HIF-1 signalling, or angiogenesis. Thus, we focused on *Vegfa*, which was the only shared gene by the GO enriched HIF-1 signalling pathway, and processes angiogenesis and differentiation. VEGFA secretion was also increased in these cells, thus further strengthening the notion of hypoxia mediated interaction between OPC and brain EC. VEGFA is a well-known regulator of angiogenesis in the CNS, which is highly expressed by neural cells including oligodendroglial cells but not by EC [52, 71]. VEGF signalling not only regulates proliferation of EC, it is also involved in the proliferation and migration of OPCs, mediated by VEGFC and VEGFA, respectively [20, 21]. This is in line with previous in vivo findings were OPC density in ischemic mice was not affected, while an increase in OPC migration in hypoxic brain areas was observed [29]. Our RNA expression data showed that when exposing in vitro OPC to hypoxia, both VEGFA expression and secretion were upregulated in a *Hif1α* and *Epas1*-dependent manner. In vivo and in vitro stabilisation of *Hif1α* in OPC have been shown to increase VEGFA expression, whereas *Hif1α* knockout or inhibition resulted in decreased VEGFA [1, 74]. The role of *Hif1α* and *Epas1* in VEGFA expression however seems to be cell specific [5, 6, 14]. Thus, we propose that in OPC, *Hif1α* is necessary for the expression of *Vegfa*, while *Epas1* has a regulatory role in hypoxic conditions. However, further investigation is needed to clarify the exact mechanism and cell-specific response involving *Hif1α* and *Epas1*. Our findings contradict previous reports that HIF stabilization in oligodendrocyte lineage does not increase *Vegfa* expression [69]. A more recent study showed that in vivo stabilisation of HIFα by *Von Hippel-Lindau* gene deletion, which is responsible for the rapid degradation of HIFα, increased *Vegfa* expression in mouse *Plp*^+^ OPC as demonstrated by in situ hybridization, which was essential for CNS angiogenesis [74]. Our in vitro work using brain EC cultured in presence of hypoxic OPC-derived CM indicates a decrease in TJ proteins, which is required for angiogenesis. Similar in vivo findings show decreased TJ proteins mRNA expression in BCAS mice, even after 3 days of hypoperfusion [34, 51, 59, 60, 76]. Together, we suggest that hypoperfusion-induced hypoxia, leading to *Hif1α* and *Epas1* stabilisation in OPC, and subsequent VEGFA production and CNS angiogenesis. Early characteristics of angiogenesis and a decrease in TJ proteins between ECs might lead to BBB dysfunction and increased BBB permeability [60, 76].

In line with the above, we then examined changes in vascular density and BBB permeability in BCAS mice. While vascular density was unaltered at 7 days of hypoperfusion, there was a significant increase in the number and size of cortical extravascular IgG levels. This indicates that 7 days of hypoperfusion does not lead to the formation of new vessels yet, but does affect BBB permeability, suggesting that BBB dysfunction is one of the first pathological structural changes occurring due to cerebral hypoperfusion. Similar results were obtained by other groups that only saw increased vascular density after 30 days, but not 7 days after BCAS, while first signs of BBB leakages were observed after 3 days [16, 38, 42, 51]. Finally, it is important to note that, while the only hypoxic glial cells identified in our in vivo study were OPC (Additional file 1: Fig. S2), our in vitro findings on VEGFA were not validated in vivo*.* Similarly, apart from IgG extravasation to assess BBB permeability in vivo, the expression of tight junction proteins was only assessed in vitro upon exposure of brain endothelial cells to conditioned medium derived from hypoxic OPC. However, a recent study showed decreased expression of CLDN5 and OCLN after 1, 3, 7, and 42 days hypoperfusion in BCAS mice, with a significant difference after 43 days compared to Sham [67]. In addition, an increased BBB permeability was observed at all timepoints in these animals [67].

Several studies have shown decreased levels of VEGFA in Alzheimer’s disease, while ischemic stroke patients have elevated levels post symptom onset [30, 45, 63, 66]. Interestingly, our results show that VEGFA plasma levels were associated with BBB leakages in the NAWM in cSVD patients, while there was no association in the WMH. This emphasises the notion that VEGFA might have a determining role in the development of vascular pathology in NAWM in an early stage, ultimately leading to the transition to WML, possibly due to subsequent neuroinflammatory reactions.

Our study presents some limitations. First, hypoxia may have affected other glial cells that could have contributed to the observed BBB dysfunction. In fact, using Cx3Cr1-GFP mice in another study, we found that microglia cells were never positive for the hypoxic marker pimonidazole (Additional file 1). However, while microglia density was not affected in BCAS vs Sham, we found that microglia acquired a pro-inflammatory phenotype in BCAS mice as shown by the increased cell area (Additional file 1: Fig. S2C) and by the increased Cx3Cr1 expression (Additional file 1: Fig. S2D). Although, microglia activation and inflammatory response in BCAS mice is well characterised, it is still largely unknown whether this is a cause or consequence of hypoperfusion and blood–brain barrier dysfunction [42, 56]. Our investigations showed that OPC were the main hypoxic glial cells affected by hypoperfusion in our BCAS model (Additional file 1: Fig. S2). This encouraged us to expose OPCs to hypoxia in vitro and to perform a transcriptomic analysis to identify key differentially expressed genes using an unbiased approach. Second, the increased VEGFA found in vitro was not validated in our in vivo model. Indeed, while the treatment of brain EC in vitro with conditioned medium derived from hypoxic OPC is valuable to assess the function in vitro, it does not help to verify the causality in our in vivo model. Therefore we suggest future studies to verify our findings in transgenic mice lacking VEGFA selectively in OPCs. Lastly, while we did find a significant correlation between VEGFA plasma concentration and BBB leakage rate in NAWM, the low *R*^*2*^ means that a considerable amount of the variance in BBB leakage is explained by other factors which we did not account for, such as increased pulsatility and impaired glymphatic drainage as a consequence of hypertension [35, 43, 48].

## Conclusion

Taken together, we suggest that cerebral hypoperfusion can lead to hypoxia in the deep cortical regions, affecting OPC, the precursors of the myelinating cells. This may trigger the production of VEGFA in hypoxic OPC via HIF1α and EPAS1 signalling, with subsequent release and action on brain EC. Our in vitro findings suggests that VEGFA may then increase the BBB permeability. This may initiate a pathophysiological cascade ultimately leading to the development of WML. It was previously hypothesised that this might be the other way around, with vascular dysfunction triggering dysfunction in OPC differentiation and myelination leading to WML [76]. Thus, future studies are needed to investigate the role of OPC-derived VEGFA in the development of WML. Understanding the OPC-vascular interaction may lead to treatment strategies specifically targeting OPC-derived VEGFA in the early development of the disease. However, this must be taken with caution, as modulating VEGFA might be a double-edged sword for its role in increasing necessary blood perfusion in hypoxic areas but also causing damage by introducing BBB leakages, thus, stressing the importance of time and cell specific targeting [70].

### Supplementary Information

**Additional file 1**.

## Data Availability

The authors confirm that the data supporting the findings of this study are available within the article and its supplementary material. Raw data are available from the corresponding author, upon request. Supplementary material is available at *Acta Neuropathologica* online.

## Abbreviations

BBB: Blood–brain barrier
BCAS: Bilateral carotid artery stenosis
CBF: Cerebral blood flow
CC: Corpus callosum
Cldn5: Claudin-5
CM: Conditioned medium
cSVD: Cerebral small vessel disease
CVON: CardioVasculair Onderzoek Nederland
DAVID: Database for annotation, visualization, and integrated discovery
DCE: Dual-time resolution dynamic contrast enhanced
DEG: Differentially expressed genes
EC: Endothelial cell
ELISA: Enzyme-linked immunosorbent assay
FBS: Fetal bovine serum
FC: Fold change
FDR: False discovery rate
FLAIR: Fluid-attenuated inversion recovery
FOV: Field of view
GO: Gene ontology
IHC: Immunohistochemistry
KEGG: Kyoto encyclopedia of genes and genomes
LDF: Laser doppler flowmetry
LSCI: Laser speckle contrast imager
MMPs: Matrix metalloproteinases
NAWM: Normal appearing white matter
NVU: Neurovascular unit
NWO: Netherlands Organisation for Scientific Research
Ocln: Occludin
OPC: Oligodendrocyte precursor cell
P/S: Penicillin/streptomycin
PFA: Paraformaldehyde
PLL: Poly-L-lysing
ROI: Region of interest
TJ: Tight junction
VEGFA: Vascular endothelial growth factor A
WM: White matter
WMH: White matter hyperintensity
WML: White matter lesion
