# Supplementary material for: Hypoxic oligodendrocyte precursor cell-derived VEGFA is associated with blood–brain barrier impairment
Source: Acta Neuropathol Commun. 2023 Aug 7;11:128. doi: 10.1186/s40478-023-01627-5 (PMC10405482; doi:10.1186/s40478-023-01627-5)
Supplement: Supplementary file 1 — Additional file 1. [file 40478_2023_1627_MOESM1_ESM.docx]

# Supplementary data

**Hypoxic oligodendrocyte precursor cell-derived VEGFA is associated with blood-brain barrier impairment**

**Narek Manukjan^1,2,3^, Daria Majcher^1^, Peter Leenders^1^, Florian Caiment^4^, Marcel van Herwijnen^4^, Hubert J. Smeets^4,5^, Ernst Suidgeest^6^, Louise van der Weerd^6,7^, Tim Vanmierlo^5,8,9^, Jacobus F.A. Jansen^5,10^, Walter H. Backes^2,5,10^, Robert J. van Oostenbrugge^2,5,11^, Julie Staals^2,11^, Daniel Fulton^3^, Zubair Ahmed^3,12,^***^†^***, W. Matthijs Blankesteijn^1,2,^***^†^***, Sebastien Foulquier^1,2,5,11,†^**

*^†^* Co-senior and co-corresponding authors

**Author affiliations:**

1. *Department of Pharmacology and Toxicology, Maastricht University, P.O. Box 616, 6200 MD Maastricht, The Netherlands*
2. *CARIM - School for Cardiovascular Diseases, Maastricht University, P.O. Box 616, 6200 MD Maastricht, The Netherlands*
3. *Neuroscience and Ophthalmology, Institute of Inflammation and Ageing, University of Birmingham, Edgbaston, Birmingham, B15 2TT, UK*
4. *Department of Toxicogenomics, GROW – School for Oncology and Developmental Biology, Maastricht University, P.O. Box 616, 6200 MD Maastricht, The Netherlands*
5. *MHeNs—School for Mental Health and Neuroscience, Maastricht University, P.O. Box 616, 6200 MD Maastricht, The Netherlands*
6. *C.J. Gorter Center for High Field MRI, Department of Radiology, Leiden University Medical Center, P.O. Box 9500, 2300 RA Leiden, the Netherlands*
7. *Department of Human Genetics, Leiden University Medical Center, P.O. Box 9500, 2300 RA Leiden, the Netherlands*
8. *Department of Neuroscience, Biomedical Research Institute, Hasselt University, 3500 Hasselt, Belgium*
9. *Department of Psychiatry and Neuropsychology, European Graduate School of Neuroscience, Maastricht University, P.O. Box 616, 6200 MD Maastricht, The Netherlands*
10. *Department of Radiology and Nuclear Medicine, Maastricht University Medical Center+, P.O. Box 5800, 6202 AZ Maastricht, The Netherlands*
11. *Department of Neurology, Maastricht University Medical Center+, P.O. Box 5800, 6202 AZ Maastricht, The Netherlands*
12. *Centre for Trauma Sciences Research, University of Birmingham, Edgbaston, Birmingham, B15 2TT, UK*

**Correspondence to:** Professor Zubair Ahmed

**Full address** Neuroscience and Ophthalmology, Institute of Inflammation and Ageing, University of Birmingham, Edgbaston, Birmingham, B15 2TT, UK

**E-mail** z.ahmed.1@bham.ac.uk

**Correspondence to:** Dr W. Matthijs Blankesteijn

**Full address** Department of Pharmacology and Toxicology, Maastricht University, 50 Universiteitssingel, 6229ER Maastricht, The Netherlands

**E-mail** wm.blankesteijn@maastrichtuniversity.nl

**Correspondence to:** Dr Sebastien Foulquier

**Full address** Department of Pharmacology and Toxicology, Maastricht University, 50 Universiteitssingel, 6229 ER Maastricht, The Netherlands

**E-mail** s.foulquier@maastrichtuniversity.nl

**Supplementary materials**

**Animals and tissue collection**

All animal experiments were approved by the regulation authority of Maastricht University and were performed in compliance with the national and European guidelines. Cx3Cr1GFP/GFP mice (Jackson Lab 005582) were crossed with Thy-1YFP/0 mice (Jackson Lab 003782) to generate Cx3Cr1GFP/WT x Thy1YFP/0 mice (abbreviated Tg mice) for microglial (GFP) and neuronal (YFP) visualization. Animals were kept on a normal 12h day-night cycle. All mice were allowed access ad libitum to water and food. Male Tg mice underwent a bilateral carotid artery stenosis (BCAS) or a Sham surgery. Briefly, microcoils with an internal diameter of 0,18mm (Sawane) were placed around the left and right common carotids in BCAS operated mice, while the same surgery was performed in Sham operated animals without placement of the microcoils. The cerebral blood flow was measured during the surgery by Laser Doppler flowmetry and was significantly decreased in BCAS vs Sham (BCAS -42±4 vs Sham +2±2, p<0,0001). BCAS operated mice were sacrificed under anaesthesia after 1 week (n=4) and 2 weeks (n=3) and Sham operated mice were sacrificed after 2 weeks (n=10). The hypoxic marker pimonidazole was injected i.p one hour before sacrifice in conscious mice (only 1week BCAS group) and a vascular tracer (70kDa-dextran-TexasRed, Thermofisher D1864, 200μL at 2,5 mg/mL) was injected i.v in isoflurane-anaesthetized mice (vena cava) 15 minutes prior to the sacrifice by decapitation. Brains were harvested directly after death without perfusion and fixed overnight in 4% paraformaldehyde (PFA). The brains were then transferred to a solution containing phosphate-buffered saline (PBS) and 1% sodium azide (NaN3) before slicing.

**Immunohistochemistry**

**Brain slicing**

Fifty μm thick coronal sections were prepared using a vibratome (VT1200S, Leica). Five consecutive series of 11-12 slices were prepared. Free-floating sections were used to stain hypoxic areas/cells (pimonidazole positive cells).

**Hypoxic cells staining**

Six identical brain slices per brain were chosen for the staining. Briefly, sections blocked in a TBS-T + 1% bovine serum albumin (BSA) solution for 2h at room temperature (RT). samples were then incubated with primary antibody overnight at 4 °C. The following day, samples were then incubated with secondary antibody and anti-pimonidazole-pacific blue antibody (Hypoxyprobe Pacific blue kit; 1:100) in TBS-T + 1% BSA for 2h at 4 °C. Finally, the slices were washed and mounted on gelatin-coated microscopic slides with a fluorescent anti-fading mounting medium (Thermofisher, Prolong gold antifade P10144).

**Image acquisition and analysis**

**Hypoxic cells and microglia**

Pimonidazole-positive cells from the corpus callosum and adjacent deep cortical and striatal areas were imaged by confocal microscopy (ACS APO 40.0 x 1.15 objective, Leica DMI 4000 microscope). Image stacks (30 μm; step size: 2 μm) including microglia (GFP) OPC (Olig2/CC1/NG2), and the hypoxic marker (Pacific blue) were acquired at a resolution of 512 x 512 pixels. Stained sections were screened for the detection of hypoxic cells within the corpus callosum and the adjacent cortical areas.

**Microglia quantification**

For the quantification of microglia density and the evaluation of morphological changes the software WIS-NeuroMath was used. Briefly, 1 μm was defined as 1,86 pixels and the settings were defined as the following: the noise level was set to 1.5, the min. cell intensity 10, min. area 10, max area 3850, min. diameter 2, max. axial ratio 10 and min. neurite length 5 μm.

**Supplementary Figure 1: Schematic overview of in vivo methods. (A)** Laser Doppler Imaging (LDI) method of CBF measurement during surgical procedures. **(B)** Laser speckle contrast imaging (LSCI) method of CBF measurements before, right after and 7 days after surgical procedures. **(C)** Bodyweight changes after surgery. **(D)** CBF changes measured with LDI during surgery. **(E)** CBF was quantified and compared to baseline measurements at d0 and d7 in the large vessels. A significant decrease of 14.7±5.3% in BCAS mice compared to baseline measurements at d0 and 27.2±3.6% at d7, with no significant changes in Sham mice at both d0 (13.1±9.0%) and d7 (10.5±5.5%). A significant difference in blood flow change between the two groups was observed at both d0 and d7. Mean±SEM; ns=not significant; #*p*<0.05, ####*p*<0.0001 vs baseline measurements; **p*<0.05, ***p*<0.01, *****p*<0.0001, vs Sham; unpaired student t-test.

**Supplementary Figure 2: Hypoxia affected the oligodendrocytes and neurons in the deep cortical regions. (A)** Immunohistochemistry for microglial (Cx3cr-GYP reporter) and hypoxic cells (Pimonidazole^+^). Scale bar, 50 µm. **(B)** 7 days of hypoperfusion did not lead to changes in microglia density, but **(C)** did acquire a pro-inflammatory phenotype in BCAS mice as shown by the increased cell area and **(D)** and by the increased Cx3Cr1 expression. **(E)** Although microglia (Cx3cr1-GFP, indicated by green arrow) were not identified as hypoxic (Pimonidazole^+^, indicated by blue arrow), the hypoperfusion induced in BCAS mice did have an impact on other glial cells (Olig2^+^, indicated by red arrow). **(F)** Hypoxic OPC were initially identified by double positive cells for OPC marker NG2 and Pimonidazole (indicated by blue arrow) **(G)** The cellular shape of pimonidazole^+^ cells (zoomed panel) that did not express Olig2 or CC1 suggested that most hypoxic cells were in fact of neuronal origin. Scale bar, 50 µm. Mean±SEM (n=3); **p*<0.05, unpaired student t-test.

**
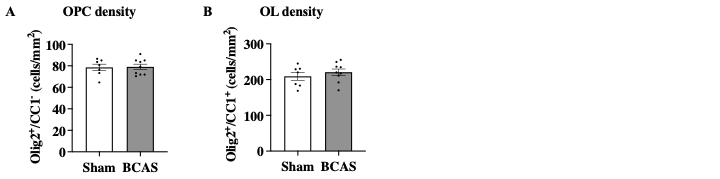
Supplementary Figure 3: BCAS does not lead to oligodendrocyte (precursor) cell density changes after 7 days.** **(A)** No differences in OPC density, quantified by the number of Olig2^+^/CC1^-^ immunolabelled cells, were found when comparing BCAS to Sham (*p*=0.91). **(B)** OL density, quantified by the number of Olig2^+^/CC1^+^ immunolabelled cells, was also unaltered in BCAS compared to Sham (*p*=0.43). Mean±SEM; unpaired student t-test.

**
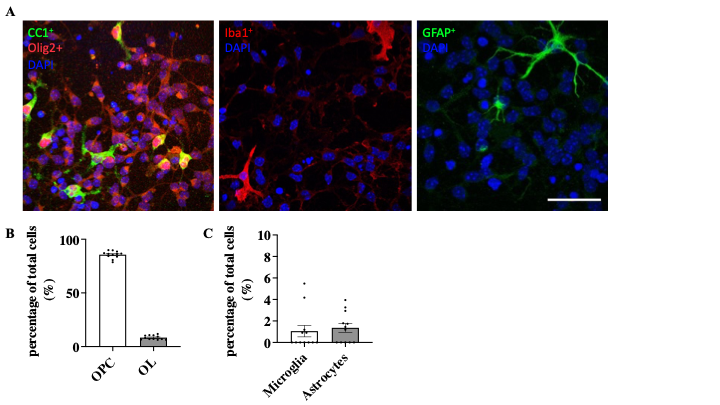
Supplementary Figure 4: Primary OPC culture purity (A)** Immunocytochemistry of primary OPC cultures. Oligodendroglial (CC1 and Olig2), microglia (Iba1), and astrocyte (GFAP) were identified in the cell cultures to determine contamination after OPC isolation from mixed glial cultures by shaking. Scale bar, 50 µm. **(B)** Quantification of number of OPC (CC1^-^/Olig2^+^, 85.6%) and mature oligodendrocytes (CC1^+^/Olig2^+^, 8.4%) as a percentage of the total number of cells (DAPI^+^). **(C)** Quantification of number of microglia (Iba1^+^, 1.1%) or astrocytes (GFAP^+^, 1.4%) as a percentage of the total number of cells (DAPI^+^). Mean±SEM.

**Supplementary Table 1:** Primary and secondary used for immunohistochemistry.

| **Primary antibody** | **Host** | **Dilution** | **Cat no.** | **Supplier** |
| --- | --- | --- | --- | --- |
| Anti-Pimonidazole conjugated to Pacific Blue fluorophore | Rat | 1:100 | HPI15 | Hypoxyprobe Inc. |
| Anti-NG2 | Rabbit | 1:250 | AB5320 | Sigma-Aldrich |
| Anti-Olig2 | Rabbit | 1:200 | AB9610 | Sigma-Aldrich |
| Anti-APC, clone CC1 | Mouse | 1:400 | MABC200 | Sigma-Aldrich |
| Anti-myelin basic protein (MBP) | Rat | 1:500 | MAB386 | Sigma-Aldrich |
| Anti-MBP, clone SMI94 | Mouse | 1:1000 | 836504 | Biolegend |
| Anti-Iba1 | Rabbit | 1:500 | 019-19741 | Wako Chemicals |
| Anti-Glial Fibrillary Acidic Protein (GFAP) | Mouse | 1:400 | G3893 | Sigma-Aldrich |
| Lycopersicon esculentum (Tomato) Lectin Texas Red | Donkey | 1:100 | TL-1176-1 | Vector Labs |
| Anti-mouse IgG Alexa Fluor 488 | Donkey | 1:200 | A-21202 | Invitrogen |
|  |  |  |  |  |
| **Secondary antibody** |  |  |  |  |
| Anti-mouse IgG Alexa Fluor 488 | Donkey | 1:200 | A-21202 | Invitrogen |
| Anti-rabbit IgG Alexa Fluor 594 | Donkey | 1:200 | A-21207 | Invitrogen |

**Supplementary Table 2:** Primers used in quantitative PCR.

| **Primer name** | **Primer sequence** |  |
| --- | --- | --- |
| *Rpl13a* | Forward | AGCCTACCAGAAAGTTTGCTTAC |
|  | Reverse | GCTTCTTCTTCCGATAGTGCATC |
| *Ywhaz* | Forward | GAAAAGTTCTTGATCCCCAATGC |
|  | Reverse | TGTGACTGGTCCACAATTCCTT |
| *Vegfa* | Forward | GCACATAGAGAGAATGAGCTTCC |
|  | Reverse | CTCCGCTCTGAACAAGGCT |
| *Hif1α* | Forward | AATGAAGTGCACCCTAACAAGCCG |
|  | Reverse | TGGCCCGTGCAGTGAAGC |
| *Epas1* | Forward | TCACTCATCCTTGCGACCAT |
|  | Reverse | TTCCCAAAACCAGAGCCGTT |
| *Cldn5* | Forward | CCACGGCCAATGGCGATTAC |
|  | Reverse | TCGTCATCCACACACGGCTT |
| *Ocln* | Forward | CCTCGGTACAGCAGCAATGG |
|  | Reverse | TAGTGGTCAGGGTCCGTCCT |

**
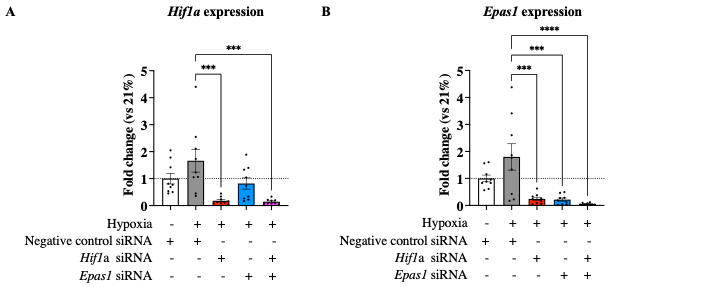
Supplementary Figure 5: Efficiency of Hif1a and Epas1 siRNA. (A)** *Hif1α* expression was significantly decreased by *Hif1α* siRNA, while *Epas1* siRNA did not significantly decrease *Hif1α* expression. **(B)** *Epas1* expression was inhibited by both *Hif1α* and *Epas1* siRNA. Treatment with both siRNA led to a significant decrease in both *Hif1α* and *Epas1* expressions. For quantification, mean±SEM; ***p<0.001, and ****p<0.0001, vs 2% + negative control siRNA; one-way ANOVA with Tukey’s multiple comparisons test.

**Supplementary Table 3: Cohort characteristics of all included participants without exclusion of identified outliers for VEGFA plasma concentration**.

|  |  | **Control (*n*=30)** | **Patient (*n*=55)** | **Univariable** | **Multivariable (age and sex corrected)** |
| --- | --- | --- | --- | --- | --- |
|  |  |  |  | **β (p-value)** | **β (p-value)** |
|  | **Age** | 68.1±2.3 | 70.4±1.4 |  |  |
|  | **Male (Female)** | 19 (11) | 35 (20) |  |  |
| **WMH** | **Relative volume, 10^-3^** | 3.5±1.6 | 15.4±2.1 | 0.391 (<0.001) | 0.402 (<0.001) |
| **BBB leakage in NAWM** | **K_i_, 10^-3^min^-1^** | 1.09±0.06 | 0.96±0.04 | -0.187 (0.086) | -0.193 (0.079) |
|  | **v_L_** | 0.298±0.034 | 0.386±0.026 | 0.215 (0.048) | 0.216 (0.050) |
| **BBB leakage in WMH** | **K_i_, 10^-3^min^-1^** | 0.87±0.05 | 0.84±0.04 | -0.048 (0.659) | -0.071 (0.531) |
|  | **v_L_** | 0.333±0.036 | 0.459±0.028 | 0.285 (0.008) | 0.281 (0.010) |

Abbreviations: WMH = white matter hyperintensities; BBB = blood brain barrier; NAWM = normal appearing white matter; K_i_ = leakage rate; v_L_ = leakage volume.

**Supplementary Table 4: Cohort characteristics of excluded participants due to measurements identified as outliers for VEGFA plasma concentration**.

|  |  | **Outliers (*n*=12)** | |
| --- | --- | --- | --- |
|  |  | **Mean±SEM** | **Min – Max** |
|  | **Age** | 73.7±2.6 | 53 – 82 |
|  | **Male (Female)** | 6 (6) |  |
| **WMH** | **Relative volume, 10^-3^** | 8.6±2.2 | 1.3 – 2.2 |
| **BBB leakage in NAWM** | **K_i_, 10^-3^min^-1^** | 0.93±0.088 | 0.6 – 1.5 |
|  | **v_L_** | 0.466±0.061 | 0.110 – 0.712 |
| **BBB leakage in WMH** | **K_i_, 10^-3^min^-1^** | 0.92±0.076 | 0.5 – 1.3 |
|  | **v_L_** | 0.449±0.061 | 0.146 – 0.739 |
| **VEGFA plasma conc.** | **pg/ml** | 405.8±121.7 | 94.9 – 1326.3 |

Abbreviations: WMH = white matter hyperintensities; BBB = blood brain barrier; NAWM = normal appearing white matter; K_i_ = leakage rate; v_L_ = leakage volume; conc. = concentration; Min = minimum; Max = maximum.

**
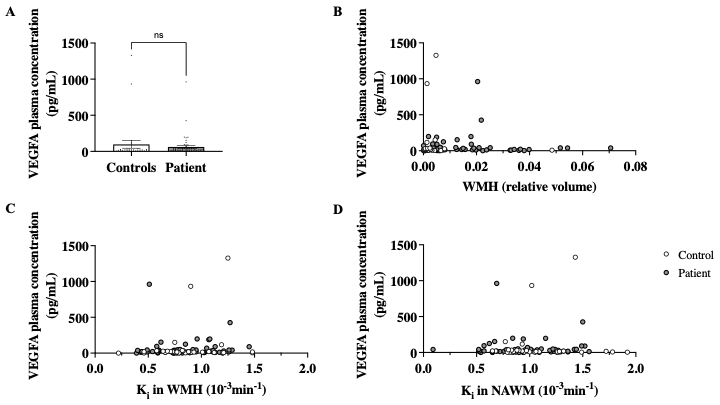
Supplementary Figure 6: No correlation between VEGFA blood plasma levels and WMH volume or leakage rate in WMH or NAWM prior to exclusion of outliers. (A)** VEGFA blood plasma levels in cSVD were not different compared to age and sex-matched controls and no correlation was seen between VEGFA plasma concentration and **(B)** WMH volume or leakage rate in **(C)** WMH and **(D)** NAWM in cSVD patients prior to exclusion of identified outliers. Abbreviations: WMH = white matter hyperintensities; NAWM = normal appearing white matter; K_i_ = leakage rate. For quantification, mean±SEM; ns = not significant; Mann-Whitney U-test.

**Supplementary Table 5: Associations between VEGFA plasma levels and MRI imaged cSVD characteristics in cSVD patients prior to exclusion of outliers**. No correlations were found between VEGFA plasma levels and sSVD parameters in the cSVD patient population prior to exclusion of outliers. P<0.05 was considered significant.

|  |  | **Univariable** | | **Multivariable** | |  |
| --- | --- | --- | --- | --- | --- | --- |
|  |  | **β** | ***p*-value** | **β** | ***p*-value** |  |
| **WMH** |  | 0.012 | 0.933 | -0.053 | 0.689 |  |
| **BBB leakage in NAWM** | **K_i_** | -0.015 | 0.913 | -0.028 | 0.846 |  |
|  |  |  |  |  |  |  |
|  | **v_L_** | -0.038 | 0.785 | -0.025 | 0.862 |  |
| **BBB leakage in WMH** | **K_i_** | -0.007 | 0.959 | -0.012 | 0.935 |  |
|  | **v_L_** | -0.048 | 0.728 | -0.038 | 0.792 |  |

Abbreviations: WMH = white matter hyperintensity; BBB = blood brain barrier; NAWM = normal appearing white matter; K_i_ = leakage rate; v_L_ = leakage volume.
